# Nurse staffing and patient care outcomes: protocol for an umbrella review to identify evidence gaps for low and middle-income countries

**DOI:** 10.12688/wellcomeopenres.17430.1

**Published:** 2021-12-23

**Authors:** Abdulazeez Imam, Sopuruchukwu Obiesie, Jalemba Aluvaala, Michuki Maina, David Gathara, Mike English

**Affiliations:** 1 Oxford Centre for Global Health Research, Nuffield Department of Medicine, University of Oxford, Oxford, OX1 3SY, UK; 2 Independent Researcher, Asaba, Nigeria; 3 KEMRI-Wellcome Trust Research Programme, Nairobi, Kenya; 4 Department of Paediatrics, University of Nairobi, Nairobi, Kenya; 5 MARCH Centre, London School of Hygiene & Tropical Medicine, London, UK

**Keywords:** Developing countries, Nurses, quality of care, patient care, ward staffing

## Abstract

**Background**: Adequate staffing is key to the delivery of nursing care and thus to improved inpatient and health service outcomes. Several systematic reviews have addressed the relationship between nurse staffing and these outcomes. Most primary studies within each systematic review are likely to be from high-income countries which have different practice contexts to low and middle-income countries (LMICs), although this has not been formally examined. We propose conducting an umbrella review to characterise the existing evidence linking nurse staffing to key outcomes and explicitly aim to identify evidence gaps in nurse staffing research in LMICs.

**Methods and analysis**: This protocol was developed using the Preferred Reporting Items for Systematic Reviews and Meta-analysis Protocols (PRISMA-P). Literature searching will be conducted across Ovid Medline, Embase and EBSCO Cumulative Index to Nursing and Allied Health Literature (CINAHL) databases. Two independent reviewers will conduct searching and data abstraction and discordance will be handled by discussion between both parties. The risk of bias of the individual studies will be performed using the AMSTAR-2 **.**

**Ethics and dissemination**: Ethical permission is not required for this review as we will make use of already published data. We aim to publish the findings of our review in peer-reviewed journals.

**PROSPERO registration number: **CRD42021286908

## Background

Globally, nurses represent almost three-fifths of all health professionals and are key to the attainment of universal health coverage ^1^. They are integral to ensuring the quality of patient care and are crucial in all health systems, playing significant roles at all levels of healthcare, including primary care where they promote mental health and well-being and anchor maternal health, growth monitoring and immunization services. They also have important roles within secondary and tertiary inpatient care settings where they plan, deliver and coordinate care and represent a critical part of the hospitals' surveillance system in detecting adverse patient events ^2^. As a result, having the right number of nurses with the right skills is paramount to sustaining any health system.

In recent times, the World Health Organization has estimated that there is a need for an extra six million nurses to actualise the global health agenda ^1^. It is estimated that around 90% of nursing shortages occur in low and middle-income countries (LMICs); particularly in Africa, South-East Asia and the eastern Mediterranean region ^1^. Only 3% of the global nurses reside in Africa, which houses 17.2% of the world’s population; this is in contrast to Europe and the Americas where 26% and 30% of global nurses reside and which have 9.6% and 17.2% share of the global population respectively ^1, 3^. The nursing crisis in LMICs represents a mismatch between increasing health service demand and the supply or employment of the nursing workforce supply ^4^. The demand is fuelled by rapid population expansion and health policies promoting universal free medical care without commensurate expansion in health services to cater for these demands ^1^. It is also driven by a rapid expansion in the scope of healthcare, changing population expectations and the increasing use of medical technologies that require more intensive nursing, for example, the use of mechanical ventilators or continuous positive airway pressure machines. The supply of nurses, on the other hand, is limited by inadequate workforce planning, financing, and investments in healthcare. The migration of highly skilled nurses from LMICs to developed countries, in search of better remuneration and improved career prospects, plays part of the role in reducing nursing levels in LMICs ^5^. It has also been suggested that LMIC source countries play a role due to limited strategies for attracting and retaining such staff ^5, 6^. Nursing shortages, while prevalent in LMICs, also occur in high-income countries (HICs) ^7^. A changing population structure resulting in an ageing population with greater healthcare needs and a steadily ageing nurse workforce, has resulting in an imbalance between nursing service demand and supply ^8^.

This imbalance in supply and demand of nurses has stimulated research on how nurse staffing might affect quality of patient care outcomes. There have been multiple studies from Europe, the U.S. and HICs, which suggest that reduced nurse staffing is associated with poorer patient outcomes ^9– 11^. Over the last two decades, several published reviews have also examined the relationship between nurse to patient ratios and the quality of hospital inpatient care ^12^. Relatively little attention has however been paid to the origins of this research, although it is likely that most studies are from HICs. As a result, the global evidence on the consequences of poor nurse staffing may mostly arise from areas with better staffing ratios. This umbrella review will examine this hypotheses.

There is a need for context specific LMIC studies or research that appraises existing evidence based on nurse staffing and quality of patient care, based on practical realities these settings. This is because staffing ratios vary across LMICs and HICs. For example, data from Kenya and Ethiopia, both LMICs report ratios can be as low as one nurse caring for 25 patients on a shift ^13, 14^, while UK Paediatric general wards report ratios of one nurse to four patients, which might approach one to one nursing in intensive care settings ^15^.

We propose an umbrella review of existing systematic reviews on nurse staffing and inpatient care. Umbrella reviews provide broader and more complete evidence on specific topics ^16^. They have also been previously shown to be an efficient way of summarising the evidence base for extensively researched topics, such as nurse staffing ^17^. Our review will pay special attention to identifying and highlighting possible evidence gaps for nurse staffing research in LMICs. It will also be important to guide the conduct of future nurse staffing research in LMICs and provide important information for policymakers.

This umbrella review aims to characterise the literature examining the consequences of nurse staffing on inpatient care and identify evidence gaps for LMICs. Our review will address the following questions:

1. What proportion of studies in published systematic reviews were conducted in LMICs?
2. What patient care outcomes do these studies report?
3. How do these outcomes differ across HICs and LMIC settings?
4. What is the range of nurse staffing levels that have been researched across acute care settings?
5. How do the nurse staffing levels described differ between LMICs and HICs?

## Methods

The protocol for this review was developed using the Preferred Reporting Items for Systematic Reviews and Meta-analysis Protocols (PRISMA-P) ^18^, and the Joanna Briggs Institute (JBI) guidelines for preparing and conducting umbrella reviews ^19^. Our review was registered with the International Prospective Register of Systematic Reviews (PROSPERO) on 27 ^th^ October 2021 (Registration number - CRD42021286908).

### Ethics

Our review is secondary research and so will not require any ethical approval. We hope to publish our findings in a peer-reviewed journal.

### Study Design

An umbrella review pulls together evidence from various systematic reviews and authors can also extract data from primary studies included in the reviews ^20^ **.** Our umbrella review will focus on systematic reviews that investigate how nurse staffing levels affect the quality of care outcomes in hospitals. It will specifically identify what evidence has been put forward for LMICs within these reviews and abstract data on the range of nurse staffing level contexts described within the primary studies of each of our included systematic reviews. We will only consider quantitative systematic reviews; qualitative, mixed-method, narrative and other umbrella reviews will be excluded from our review, as well as commentaries, editorials, and review protocols. LMIC in this paper will be defined using the World Bank’s country and lending groups classification system, which classifies 135 countries into low-income, low-middle-income and upper-middle-income economies, based on gross national income per capita.

### Population

We will include systematic reviews where the focus was on patients admitted to hospital ward settings which might either include newborn, paediatric or adult wards. We will however exclude systematic reviews of intensive care units, as the staffing of these units varies significantly from regular ward care settings, which is the focus of our umbrella review. We will also exclude systematic reviews of non-hospital settings, such as community clinics and nursing homes or settings where care is not carried out continuously, such as outpatient clinics. For those systematic reviews that combine both studies conducted in intensive care units and hospital ward settings, we will include them but only report on the primary studies that were conducted in ward care settings.

### Exposure

Our exposure of interest is nurse staffing and its effect on patient care outcomes. Our review will consider a wide variety of staffing metrics reported by authors. This includes but is not limited to, nurse-to-patient ratios, nurse-to-bed ratios, or nursing hour per patient days. Our focus will be to identify systematic reviews that investigate the impact of any of these staffing metrics on patient outcomes. We will exclude systematic reviews which investigated the impact of other staffing metrics, for example, nursing skill mix and nursing work schedules on patient care outcomes.

### Outcome

Our primary outcome of interest will be the quality of patient care. This includes patient care outcomes described in systematic reviews, for example length of hospital stays and the incidences of hospital-acquired infection and mortality. We will also consider nursing process outcomes such as missed nursing care or errors in administering medications. For reviews that report mixed outcomes, for example reviews on nurse and patient outcomes, we will include them but only report on their patient care outcomes.

### Setting

We aim to identify the broad range of quality-of-care outcomes studied across systematic reviews and identify how these reported outcomes differ between HICs and LMICs. We will also identify the range of staffing levels, where individual studies reported in the reviews have been conducted and we will compare these across LMIC and HICs.

### Search strategy

We will conduct our search in databases for systematic reviews, these include the Cochrane Register of Systematic Reviews, the JBI Database of Systematic Reviews and Implementation Reports and other databases such as Medline, Embase and Cumulative Index to Nursing and Allied Health Literature (CINAHL). The databases will be filtered by, to only show reviews published in English, due to limitations in translation. There will be no date restrictions applied to our search and we will identify reviews published from inception of the databases till when we conduct our searches. We will also perform hand searches in some select journals and search the reference list of articles we identify for additional papers. We contacted a health information librarian to develop our search strategy, and this was piloted in Ovid Medline (see *Extended data* ^21^). We also conducted initial searches in Prospero to identify ongoing or planned reviews that might relate to our proposed research before undertaking the review.

### Data management

Following our search, we will upload all retrieved abstracts of the identified systematic reviews to Zotero, a reference management software, where we will perform deduplication ^22^. Following this, titles and abstracts of the retrieved papers will be screened for relevance by two independent reviewers using the Rayyan – Intelligent Systematic Review, a web-based application for screening ^23^. Full text of potentially relevant articles will then be scrutinised by both reviewers independently using the pre-defined inclusion criteria. In the event of any discordance, this will be resolved through discussion and if unsuccessful, arbitration by a third reviewer.

### Data items

Pre-review, we will develop a standardised Microsoft Excel form to extract data on each of the identified systematic reviews for our umbrella review. This will include publication year, first author’s name, outcome type/types measured, the number of studies included in each identified review that reported on specific patient outcomes in regular ward settings, and countries where these studies were conducted. This will be used to determine the proportion of studies conducted in LMIC settings and if a difference exists in outcomes studied in both LMICs and HICs. We will also extract the type of staffing metrics reported by the individual studies within each review, and a summary statistic describing this, from the study summary table, provided in each of our eligible reviews. If this data is unavailable or incomplete, we will abstract this from the primary articles of the selected systematic reviews. The range of staffing will be our preferred metric. In the event there is no range specified, the mean or median staffing metric will be preferred. For a staffing metric to be reported, the parent study should report one of our previously identified nurse staffing metrics and determine its association with any patient care outcomes in a regular ward setting.

### Quality assessment

We will use the AMSTAR-2 criteria to assess the methodological quality of each systematic review ^24^. This is a widely used tool for appraising systematic reviews and is recommended by the JBI guidelines for preparing and conducting umbrella reviews ^19^. Assessments will be carried out independently by two reviewers, AI and SO. Any disagreements will be managed through discussions and in event of a lack of consensus, a third reviewer will be invited as a tiebreaker. As we aim for our umbrella review to provide broad information on patient care outcomes in LMICs, we will include all eligible systematic reviews in our synthesis irrespective of their risk of bias scores, but we will discuss any potential impact of these scores in our evidence synthesis.

### Data synthesis

The findings of our umbrella review will be reported using a narrative synthesis as we do not include any effect estimates. We will summarise each review, providing details on the research context, period of review, objectives and primary studies included in the review.

We intend to group studies conducted within each systematic review into those conducted in LMIC settings and those in non-LMIC settings using the World Bank’s country and lending group classification system. Our data synthesis will describe the proportion of LMIC studies within each systematic review. We will also compare the broad range of patient care outcomes described across both LMIC and HIC settings and the range of staffing levels in both care settings. Our data will be presented using a combination of tables, figures, and clustered bar charts.

### Study status

We confirm that at the time of submission of this protocol, we have completed full-text screening of identified articles.

## Discussion

Nurse staffing has long been recognised as one of the key factors that affect the quality of patient care. While staffing requirements might vary across different health care settings, HICs have traditionally had better staffing-to-patient ratios compared to many LMICs. Additionally, more research into staffing and patient care outcomes is conducted in HICs and these studies might form the bulk of evidence for systematic reviews in this area. Our Umbrella review determines the extent of this and proposes to identify the evidence gaps for LMICs in terms of contributions to the overall literature on nurse staffing and quality of patient care.

### Data availability

No underlying data are associated with this article.

### Extended data

Open Science Framework: Nurse staffing and patient care outcomes: protocol for an umbrella review to identify evidence gaps for low and middle-income countries https://doi.org/10.17605/OSF.IO/7YTKX ^21^

This project contains the following extended data:

- Search strategy for Ovid Medline.docx

### Reporting guidelines

Open Science Framework: PRISMA-P checklist for ‘Nurse staffing and patient care outcomes: protocol for an umbrella review to identify evidence gaps for low and middle-income countries’. https://doi.org/10.17605/OSF.IO/7YTKX ^21^

Data are available under the terms of the Creative Commons Attribution 4.0 International license (CC-BY 4.0).
