# Nurse staffing and patient care outcomes: protocol for an umbrella review to identify evidence gaps for low and middle-income countries in global literature

**DOI:** 10.12688/wellcomeopenres.17430.2

**Published:** 2022-05-12

**Authors:** Abdulazeez Imam, Sopuruchukwu Obiesie, Jalemba Aluvaala, Michuki Maina, David Gathara, Mike English

**Affiliations:** 1 Oxford Centre for Global Health Research, Nuffield Department of Medicine, University of Oxford, Oxford, OX1 3SY, UK; 2 Independent Researcher, Asaba, Nigeria; 3 KEMRI-Wellcome Trust Research Programme, Nairobi, Kenya; 4 Department of Paediatrics, University of Nairobi, Nairobi, Kenya; 5 MARCH Centre, London School of Hygiene & Tropical Medicine, London, UK

**Keywords:** Developing countries, Nurses, quality of care, patient care, ward staffing

## Abstract

**Background**: Adequate staffing is key to the delivery of nursing care and thus to improved inpatient and health service outcomes. Several systematic reviews have addressed the relationship between nurse staffing and these outcomes. Most primary studies within each systematic review are likely to be from high-income countries which have different practice contexts to low and middle-income countries (LMICs), although this has not been formally examined. We propose conducting an umbrella review to characterise the existing evidence linking nurse staffing to key outcomes and explicitly aim to identify evidence gaps in nurse staffing research in LMICs.

**Methods and analysis**: This protocol was developed using the Preferred Reporting Items for Systematic Reviews and Meta-analysis Protocols (PRISMA-P). Literature searching will be conducted across Ovid Medline, Embase and EBSCO Cumulative Index to Nursing and Allied Health Literature (CINAHL) databases. Two independent reviewers will conduct searching and data abstraction and discordance will be handled by discussion between both parties. The risk of bias of the individual studies will be performed using the AMSTAR-2 **.**

**Ethics and dissemination**: Ethical permission is not required for this review as we will make use of already published data. We aim to publish the findings of our review in peer-reviewed journals.

**PROSPERO registration number: **CRD42021286908

## Background

Globally, nurses represent almost three-fifths of all health professionals and are key to the attainment of universal health coverage ^1^. They are integral to ensuring the quality of patient care and are crucial in all health systems, playing significant roles at all levels of healthcare, including primary care where they promote mental health and well-being and anchor maternal health, growth monitoring and immunization services. They also have important roles within secondary and tertiary inpatient care settings where they plan, deliver and coordinate care and represent a critical part of the hospitals' surveillance system in detecting adverse patient events ^2^. As a result, having the right number of nurses with the right skills is paramount to sustaining any health system.

In the year 2020, the World Health Organization has estimated that there was a need for an extra six million nurses to actualise the global health agenda ^1^. It is estimated that around 90% of nursing shortages occur in low and middle-income countries (LMICs); particularly in Africa, South-East Asia, and the eastern Mediterranean region ^1^. Only 3% of the global nurses reside in Africa, which houses 17.2% of the world’s population and where there is an ever increasing non-communicable disease epidemic which requires long term care and for which nurses are critical ^1, 3, 4^. The nursing crisis in LMICs represents a mismatch between increasing health service demand and the supply or employment of the nursing workforce supply ^5^. The demand is fuelled by rapid population expansion and health policies promoting universal free medical care without commensurate expansion in health services to cater for these demands ^1^. It is also driven by a rapid expansion in the scope of healthcare, changing population expectations and the increasing use of medical technologies that require more intensive nursing, for example, the use of mechanical ventilators or continuous positive airway pressure machines. The supply of nurses, on the other hand, is limited by inadequate workforce planning, financing, and investments in healthcare. The migration of highly skilled nurses from LMICs to developed countries, in search of better remuneration and improved career prospects, plays part of the role in reducing nursing levels in LMICs ^6^. It has also been suggested that LMIC source countries play a role due to limited strategies for attracting and retaining such staff ^6, 7^. 

This imbalance in supply and demand of nurses has stimulated research on how nurse staffing might affect quality of patient care outcomes but these have come mainly from HICs which have distinctively different organisational contexts and comparatively better staffing ratios than LMICs ^8– 10^. The relative extent and nature of the evidence gaps for LMICs in the global literature for nurse staffing and quality of care has not been formally examined.

An Umbrella review (an overview of systematic reviews) provides broad and complete evidence on specific topics by synthesising the information across multiple systematic reviews ^11^. This is also seen as a more efficient way of synthesising data from areas of research where a large amount of research might have been conducted ^11^. Over the last two decades, several published reviews have examined the relationship between nurse staffing and the quality of hospital inpatient care ^12– 16^. A synthesis of these reviews examining the global evidence with special attention paid to identifying and highlighting possible evidence gaps for nurse staffing research in LMICs would be highly informative to highlighting the evidence gaps for LMICs. This would be relevant to guide the future conduct of nurse staffing research in LMIC settings and would be important for policymakers who are involved in defining nurse staffing in these areas.

Our umbrella review thus aims to characterise the literature examining the consequences of nurse staffing on inpatient care and identify evidence gaps for LMICs. Our review will aim to:

1. Identify the origins of studies within published reviews and determine what proportion of these were conducted in LMICs?
2. Identify the patient care outcomes reported across reviews and determine how reported outcomes differ across HICs and LMICs?
3. Describe the range of nurse staffing levels that have been researched across acute care settings and determine how these differ between LMICs and HICs?

## Methods

The protocol for this review was developed using the Preferred Reporting Items for Systematic Reviews and Meta-analysis Protocols (PRISMA-P) ^17^, and the Joanna Briggs Institute (JBI) guidelines for preparing and conducting umbrella reviews ^11^. Our review was registered with the International Prospective Register of Systematic Reviews (PROSPERO) on 27 ^th^ October 2021 (Registration number - CRD42021286908).

### Ethics and dissemination

Our review is secondary research and so will not require any ethical approval. We hope to publish our findings in a peer-reviewed journal.

### Study design

An umbrella review pulls together evidence from various systematic reviews and authors can also extract data from primary studies included in the reviews ^18^. Our umbrella review will focus on systematic reviews that investigate how nurse staffing levels affect the quality of care outcomes in hospitals. It will appraise the existing evidence within these reviews and specifically highlight what evidence has been put forward for LMICs in comparison to HICs. We will also abstract data on the range of nurse staffing level contexts described within the primary studies of each of our included systematic reviews. We will only consider quantitative systematic reviews; qualitative, mixed-method, narrative and other umbrella reviews will be excluded from our review, as well as commentaries, editorials, and review protocols. LMIC in this paper will be defined using the World Bank’s country and lending groups classification system, which classifies 135 countries into low-income, low-middle-income and upper-middle-income economies, based on gross national income per capita as at 23 ^rd^ December, 2021.

### Population

We will include systematic reviews where the focus was on patients admitted to hospital ward settings which might either include newborn, paediatric or adult wards. We will however exclude systematic reviews of intensive care units, as the staffing ratios of these units tend to be better than regular ward care settings ^19^, which is the focus of our umbrella review. We will also exclude systematic reviews of non-hospital settings, such as community clinics and nursing homes or settings where care is not carried out continuously, such as outpatient clinics. For those systematic reviews that combine both studies conducted in intensive care units and hospital ward settings, if feasible, we will include them but only report on the primary studies that were conducted in ward care settings.

### Exposure

Our exposure of interest is nurse staffing and its effect on patient care outcomes. Our review will consider a wide variety of staffing metrics reported by authors. This includes but is not limited to, nurse-to-patient ratios, nurse-to-bed ratios, or nursing hour per patient days. Our focus will be to identify systematic reviews that investigate the impact of any of these staffing metrics on patient outcomes. We will exclude systematic reviews which investigated the impact of other nursing metrics, for example, nursing skill mix and nursing work schedules on patient care outcomes, as the focus of this umbrella review is on the effects of variation nurse number or time available related to nurse staffing rather than how existing nurse numbers are organised.

### Outcome

Our primary outcome of interest will be the quality of patient care. This includes patient care outcomes described in systematic reviews, for example length of hospital stays and the incidences of hospital-acquired infection and mortality. We will also consider nursing process outcomes such as missed nursing care or errors in administering medications. For reviews that report mixed outcomes, for example reviews on nurse and patient outcomes, we will include them but only report on their patient care outcomes.

### Setting

We aim to identify the broad range of quality-of-care outcomes studied across systematic reviews and identify how these reported outcomes differ between HICs and LMICs. We will also identify the range of staffing levels, where individual studies reported in the reviews have been conducted and we will compare these across LMIC and HICs.

### Search strategy

We will conduct our search in databases for systematic reviews, these include the Cochrane Register of Systematic Reviews, the JBI Database of Systematic Reviews and Implementation Reports and other databases such as Medline, Embase and Cumulative Index to Nursing and Allied Health Literature (CINAHL). The databases will be filtered by, to only show reviews published in English, due to limitations in translation capacity among the research team. There will be no date restrictions applied to our search and we will identify reviews published from inception of the databases till when we conduct our searches. We will also perform hand searches in some select journals and search the reference list of articles we identify for additional papers. 

Prior to commencing the review, weconducted initial searches in Prospero to identify ongoing or planned umbrella reviews that might be similar to ours. We contacted a health information librarian to develop our search strategy, and this was piloted in Ovid Medline (see *Extended data* ^20^). We identified our search terms by identifying repeated keywords and MESH terms of some pre-identified papers in Scopus and searching for synonyms of these.

### Data management

Following our search, we will upload all retrieved abstracts of the identified systematic reviews to Zotero, a reference management software, where we will perform deduplication ^21^. Following this, titles and abstracts of the retrieved papers will be screened for relevance by two independent reviewers using the Rayyan – Intelligent Systematic Review, a web-based application for screening ^22^. Full text of potentially relevant articles will then be scrutinised by both reviewers independently using the pre-defined inclusion criteria. In the event of any discordance, this will be resolved through discussion and if unsuccessful, arbitration by a third reviewer.

### Quality assessment

We will use the AMSTAR-2 criteria to assess the methodological quality of each systematic review ^23^. This is a widely used tool for appraising systematic reviews and is recommended by the JBI guidelines for preparing and conducting umbrella reviews ^11^. Assessments will be carried out independently by two reviewers, AI and SO. Any disagreements will be managed through discussions and in event of a lack of consensus, a third reviewer will be invited as a tiebreaker. As we aim for our umbrella review to provide broad information on patient care outcomes in LMICs, we will include all eligible systematic reviews in our synthesis irrespective of their risk of bias scores, but we will discuss any potential impact of these scores in our evidence synthesis.

### Data items

We developed a standardised Microsoft Excel form to extract data on each of the identified systematic reviews for our umbrella review. This included publication year, first author’s surname, outcome type/types measured, the number of studies included in each identified review that reported on specific patient outcomes in regular ward settings, and countries where these studies were conducted. This will be used to determine the proportion of studies conducted in LMIC settings and if a difference exists in outcomes studied in both LMICs and HICs. To address our objective on the range of nurse staffing reported across care settings, we will also extract the type of staffing metrics reported by the individual studies within each review, and a summary statistic describing this. We will abstract this from the study summary table, provided in each of our eligible reviews. If this data is unavailable or incomplete, we will retrieve this from the primary articles of the selected systematic reviews. The range of staffing will be our preferred metric. In the event there is no range specified, the mean or median staffing metric will be preferred.

### Data synthesis

The findings of our umbrella review will be reported using a narrative synthesis as we do not include any effect estimates ^24^. We will summarise each review, providing details on the research context, period of review, objectives and primary studies included in the review.

We intend to group studies conducted within each systematic review into those conducted in LMIC settings and those in non-LMIC settings using the World Bank’s country and lending group classification system. Our data synthesis will describe the proportion of LMIC studies within each systematic review. We will also compare the broad range of patient care outcomes described across both LMIC and HIC settings and the range of staffing levels in both care settings. Our data will be presented using a combination of tables and figures.

### Study status

We confirm that at the time of submission of this protocol, we have completed full-text screening of identified articles.

## Discussion

Nurse staffing has long been recognised as one of the key factors that affect the quality of patient care. While staffing requirements might vary across different health care settings, HICs have traditionally had better staffing-to-patient ratios compared to many LMICs. Additionally, more research into staffing and patient care outcomes is conducted in HICs and these studies might form the bulk of evidence for systematic reviews in this area. Such research has shaped Human Resource for Health policies particularly in nurse staffing in HICs, where for example, UK Paediatric general wards report ratios of one nurse to four patients, which might approach one to one nursing in intensive care ^25^. In contrast, studies from Ethiopia and Kenya, both LMICs report ratios as low as one nurse caring for 25 patients on a shift ^26, 27^. The evidence for nurse staffing in LMICs is unclear and data to guide this is limited ^15^.

Pre-review we identified one systematic review which focused on nurse staffing and patient care outcomes in LMICs that demonstrated poor quality evidence ^15^. Our Umbrella review builds on this earlier work my appraising the evidence for LMICs in the context of global literature and identifying evidence gaps for such settings through comparisons made with HICs. This is an essential first step to mapping out areas for future LMIC research.

## Data availability

No underlying data are associated with this article.

### Extended data

Open Science Framework: Nurse staffing and patient care outcomes: protocol for an umbrella review to identify evidence gaps for low and middle-income countries https://doi.org/10.17605/OSF.IO/ZYE7X ^20^

This project contains the following extended data:

- Search strategy for Ovid Medline.docx
- Search strategy for Embase
- Search strategy for CINAHL

### Reporting guidelines

Open Science Framework: PRISMA-P checklist for ‘Nurse staffing and patient care outcomes: protocol for an umbrella review to identify evidence gaps for low and middle-income countries’. https://doi.org/10.17605/OSF.IO/ZYE7X ^20^

Data are available under the terms of the Creative Commons Attribution 4.0 International license (CC-BY 4.0).
