# Supplementary material for: Nurse staffing and patient care outcomes: protocol for an umbrella review to identify evidence gaps for low and middle-income countries in global literature
Source: Wellcome Open Res. 2022 May 12;6:363. Originally published 2021 Dec 23. [Version 2] doi: 10.12688/wellcomeopenres.17430.2 (PMC8943346; doi:10.12688/wellcomeopenres.17430.2)
Supplement: Peer review history

# Peer review: Reviewer response for version 2

**DOI:** 10.21956/wellcomeopenres.19774.r50498

**Published:** 2022-05-17

**Authors:** Aduragbemi Banke-Thomas

**Affiliations:** 1 School of Human Sciences, University of Greenwich, London, UK

Thanks to the authors for taking the time to address my comments in the earlier version painstakingly. These have now all been resolved and I wish them the very best in the actual review.

Is the study design appropriate for the research question?

Yes

Is the rationale for, and objectives of, the study clearly described?

No

Are sufficient details of the methods provided to allow replication by others?

Yes

Are the datasets clearly presented in a useable and accessible format?

Not applicable

Reviewer Expertise:

Maternal Health; Health Policy

I confirm that I have read this submission and believe that I have an appropriate level of expertise to confirm that it is of an acceptable scientific standard.

# Peer review: Reviewer response for version 2

**DOI:** 10.21956/wellcomeopenres.19774.r50497

**Published:** 2022-05-13

**Authors:** Uzochukwu Egere

**Affiliations:** 1 Department of International Public Health, Liverpool School of Tropical Medicine, Liverpool, UK

I am happy with the amendments the authors have made on this manuscript, and am happy for it to be indexed in its current form.

Is the study design appropriate for the research question?

Yes

Is the rationale for, and objectives of, the study clearly described?

Yes

Are sufficient details of the methods provided to allow replication by others?

Yes

Are the datasets clearly presented in a useable and accessible format?

Not applicable

Reviewer Expertise:

Clinical medicine (Paediatrics), Public health, Implementation research, Health systems analysis, Health services research.

I confirm that I have read this submission and believe that I have an appropriate level of expertise to confirm that it is of an acceptable scientific standard.

# Peer review: Reviewer response for version 1

**DOI:** 10.21956/wellcomeopenres.19273.r49080

**Published:** 2022-03-23

**Authors:** Uzochukwu Egere

**Affiliations:** 1 Department of International Public Health, Liverpool School of Tropical Medicine, Liverpool, UK

General comments:

 Thanks for asking me to review this protocol which details an umbrella review to characterize the existing evidence linking nurse staffing to key outcomes, and also identifies and highlights possible evidence gaps for nurse staffing research in low- and middle-income countries (LMICs). The authors make a strong case for conducting this review and draw on several important publications to bring more clarity into the subject.

 At this time of renewed determination to ensure universal health coverage especially in the vulnerable LMIC settings, this umbrella review has a huge potential to impact policy and inform strategies for health systems strengthening. 

 I would like to see data from LMICs disaggregated by countries and health system levels. Though there are huge nursing staff shortages in these settings, the shortages are likely to vary widely across health systems levels. I suggest that the authors consider these data items during data extraction.

Specific comments:

 Background: The data on percentage of global nurses resident in each region versus the population they serve, is helpful. However, I wonder if this ratio would change for Latin America, for instance, if the USA is taken out of the 'Americas' region. Secondly, the discussion on the demand and supply crisis could also highlight intra-country dynamics such as movements from rural to urban areas as contributory to the crisis within LMICs.

 Study design: It is in order to focus on quantitative variables as the authors have done here, given the review questions. It may therefore be necessary to consider abstracting quantitative data from the mixed methods studies instead of completely excluding all mixed methods studies. 

 Population: The authors mention in the review questions that they will find out the "range of nurse staffing levels that have been researched across acute care settings" but then in this (Population) section, it mentioned that systematic reviews of intensive care units will be excluded. It might be in order to clarify what is meant by "acute care settings" in this review.

Is the study design appropriate for the research question?

Yes

Is the rationale for, and objectives of, the study clearly described?

Yes

Are sufficient details of the methods provided to allow replication by others?

Yes

Are the datasets clearly presented in a useable and accessible format?

Not applicable

Reviewer Expertise:

Clinical medicine (Paediatrics), Public health, Implementation research, Health systems analysis, Health services research.

I confirm that I have read this submission and believe that I have an appropriate level of expertise to confirm that it is of an acceptable scientific standard.

# Peer review: Reviewer response for version 1

**DOI:** 10.21956/wellcomeopenres.19273.r49078

**Published:** 2022-03-15

**Authors:** Aduragbemi Banke-Thomas

**Affiliations:** 1 School of Human Sciences, University of Greenwich, London, UK

Thank you for inviting me to review this protocol. I read it with a lot of interest.

 I have suggested a number of points that I believe will help to really strengthen the protocol. The two big comments point to the need to provide a stronger rationale underpinning the need for this umbrella review. This needs to come out very clearly. Second is there is a need to clarify if the focus of this review is purely LMIC or if it is global. I think it is global, that wants to show there is an evidence gap for LMICs. I would argue that the protocol needs to reflect this.

 Specific points are presented below:

- The title as written, "Nurse staffing and patient care outcomes: protocol for an umbrella review to identify evidence gaps for low and middle-income countries" might not be appropriate as you still collect data from HICs to identify gaps. This is more of a global review. Your review questions certainly do not suggest that this is a purely LMIC focused review. Please tweak the title to reflect the focus.
- Please specify the period when you write "In recent times, the World Health Organization...".
- For this statement, "Only 3% of the global nurses reside in Africa, which houses 17.2% of the world’s population..". Rather than relating this to the population, please relate to the burden of disease, especially NCDs that need long term care for which nurses are particularly critical.
- Kindly motivate the introduction better to improve flow. This statement, "Nursing shortages, while prevalent in LMICs, also occur in high-income countries (HICs)..." shows the global reach of this planned review. Please structure the section in a way that shows the LMIC and HIC foci.
- A stronger case for this review of reviews is needed. You write that "Umbrella reviews provide broader and more complete evidence on specific topics ^16^. They have also been previously shown to be an efficient way of summarising the evidence base for extensively researched topics, such as nurse staffing ^1". ^Can you evidence that nurse staffing is an example of an "extensively researched topic"? Yet you make a point already that this topic is minimally researched in LMICs. If it is not well sufficiently studied in LMICs, then a review of LMIC original papers will do. Summary: The rationale for an umbrella review does not come out clearly. Please review and rephrase.
- Ref 16 will be better as a methodology paper that describes the process of an umbrella review not an example of another one. Maybe consider using Ref 19 and deleting 16.
- You have cited JBI guidelines for umbrella reviews, registered with PROSPERO, and leveraged PRISMA-P. Good!
- This statement "We hope to publish our findings in a peer-reviewed journal." has nothing to do with ethics. It is more about dissemination. Please remove or move elsewhere.
- Please align your methods to your research question and frankly the entire protocol (title, background and methods). Under study design, you only talk about LMIC. Elsewhere, you talk about LMIC and HIC. This is difficult to follow. Please be more consistent in this protocol.
- The World Bank classification is fluid. Be specific with cut off date of definition where you write "LMIC in this paper will be defined using the World Bank’s country and lending groups classification system, which classifies 135 countries into low-income, low-middle-income and upper-middle-income economies, based on gross national income per capita".
- Please use evidence to justify your exclusion criteria. For example, you write, "We will however exclude systematic reviews of intensive care units, as the staffing of these units varies significantly from regular ward care settings...".
- These sort of protocols typically include their detailed search strategy across multiple databases, so this will not be needed with the same level of detail in the actual review. Please include the detailed search strategy across the different databases to be searched.
- It might also be helpful to show how you have actually reached the search terms used. So, in essence, detail the piloting of the search strategy.
- Not sure if this will be possible in all cases "For those systematic reviews that combine both studies conducted in intensive care units and hospital ward settings". Please rethink.
- You write, "We will exclude systematic reviews which investigated the impact of other staffing metrics, for example, nursing skill mix and nursing work schedules on patient care outcomes". Please explain why.
- This is not clear, "...due to limitations in translation". Do you mean no services available? Or no one on the team can translate? Please think about this a bit more. Are there actually papers that you will really be missing?
- This sentence is consuming "We also conducted initial searches in Prospero to identify ongoing or planned reviews that might relate to our proposed research before undertaking the review.". Was this done to find out if another team is doing an umbrella review? Or if there are ongoing reviews of primary studies? If former, then this should be moved upwards. Either way, this needs to be rephrased for clarity.
- You write, "Pre-review, we will develop a standardised Microsoft Excel form to extract data on each of the identified systematic reviews for our umbrella review". Should this not be available already?
- Should quality assessment come before data extraction?
- Please review the content being extracted from the reviews. You write "first author’s name". Is it the name or surname or full name? It is not clear why the metrics such as nurse-to-patient ratios, nurse-to-bed ratios, or nursing hour per patient days need to be extracted from the primary studies. Do you want to maybe review this and fix it from the point of inclusion? 
- Please cite the approach being used for synthesis - narrative synthesis.
- Clustered bar charts are also Figures. Is there a reason why this is being specified?
- Please rewrite the discussion to reflect the global nature of this review of reviews. In addition, make a case for why it is important to conduct this review of reviews. What will it show differently compared to what the actual reviews will not show? You can also bring in your dissemination plan (which I suggested should be deleted from ethics above) here.

I hope you find these comments useful and look forward to the revised version. Very best wishes.

Is the study design appropriate for the research question?

Yes

Is the rationale for, and objectives of, the study clearly described?

No

Are sufficient details of the methods provided to allow replication by others?

Yes

Are the datasets clearly presented in a useable and accessible format?

Not applicable

Reviewer Expertise:

Maternal Health; Health Policy

I confirm that I have read this submission and believe that I have an appropriate level of expertise to confirm that it is of an acceptable scientific standard, however I have significant reservations, as outlined above.

# Response

**Published:** 2022-04-14

**Authors:** Abdulazeez Imam

**Affiliations:** University of Oxford, UK

Reviewer: 2

 [Aduragbemi Banke-Thomas, School of Human Sciences, University of Greenwich, London, UK ]

 We thank the reviewer for making time to read our manuscript and providing invaluable feedback. We have responded to all their comments and our responses come in italics under each specific comment

 Comment 1: The title as written, "Nurse staffing and patient care outcomes: protocol for an umbrella review to identify evidence gaps for low and middle-income countries" might not be appropriate as you still collect data from HICs to identify gaps. This is more of a global review. Your review questions certainly do not suggest that this is a purely LMIC focused review. Please tweak the title to reflect the focus.

*Thank you for this comment. We recognise the reviewer’s concerns have now modified the title to: “ **Nurse staffing and patient care outcomes: protocol for an umbrella review to identify evidence gaps for low and middle-income countries in global literature”.***

 Comment 2: Please specify the period when you write "In recent times, the World Health Organization

*We thank the reviewer for this comment and have now stated this. Please see introduction line 11.*

 Comment 3: For this statement, "Only 3% of the global nurses reside in Africa, which houses 17.2% of the world’s population..". Rather than relating this to the population, please relate to the burden of disease, especially NCDs that need long term care for which nurses are particularly critical.

*We thank the reviewer for this comment. We have now modified the introduction to include a statement relating the nursing crisis in LMICs to the burden of NCDs. Introduction lines 14 to 17.*

 Comment 4: Kindly motivate the introduction better to improve flow. This statement, "Nursing shortages, while prevalent in LMICs, also occur in high-income countries (HICs)..." shows the global reach of this planned review. Please structure the section in a way that shows the LMIC and HIC foci.

*We thank the reviewer for this comment and have now restructured our introduction*

 5: A stronger case for this review of reviews is needed. You write that "Umbrella reviews provide broader and more complete evidence on specific topics ^16^ . They have also been previously shown to be an efficient way of summarising the evidence base for extensively researched topics, such as nurse staffing ^1^ ^". ^Can you evidence that nurse staffing is an example of an "extensively researched topic"? Yet you make a point already that this topic is minimally researched in LMICs. If it is not well sufficiently studied in LMICs, then a review of LMIC original papers will do. Summary: The rationale for an umbrella review does not come out clearly. Please review and rephrase.

*We thank the reviewer for this comment. We have re-written this section to strengthen the rationale for an umbrella review. Please see the introduction, lines 35 to 45.*

 Comment 6:  Ref 16 will be better as a methodology paper that describes the process of an umbrella review not an example of another one. Maybe consider using Ref 19 and deleting 16

*This has now been modified.*

 Comment 7: You have cited JBI guidelines for umbrella reviews, registered with PROSPERO, and leveraged PRISMA-P. Good!

*Thank you.*

 Comment 8: This statement "We hope to publish our findings in a peer-reviewed journal." has nothing to do with ethics. It is more about dissemination. Please remove or move elsewhere.

*We thank the reviewer for this comment and have now edited this section to Ethics and dissemination.*

**

 Comment 9: Please align your methods to your research question and frankly the entire protocol (title, background, and methods). Under study design, you only talk about LMIC. Elsewhere, you talk about LMIC and HIC. This is difficult to follow. Please be more consistent in this protocol

*We thank the reviewer for this comment, and we have now checked we are consistent throughout the paper. We have edited our title and objectives to also reflect the global nature of the review.*

 Comment 10: The World Bank classification is fluid. Be specific with cut off date of definition where you write "LMIC in this paper will be defined using the World Bank’s country and lending groups classification system, which classifies 135 countries into low-income, low-middle-income and upper-middle-income economies, based on gross national income per capita"

*We thank the reviewer for this comment, and we have now amended this.*

 Comment 11: Please use evidence to justify your exclusion criteria. For example, you write, "We will however exclude systematic reviews of intensive care units, as the staffing of these units varies significantly from regular ward care settings..."

*We thank the reviewer for this comment, and we have now amended this. Please see population, lines 81 to 83.*

 Comment 12: These sorts of protocols typically include their detailed search strategy across multiple databases, so this will not be needed with the same level of detail in the actual review. Please include the detailed search strategy across the different databases to be searched

*We thank the reviewer for this comment, and we have included these searches in the extended data.*

 Comment 13: It might also be helpful to show how you have actually reached the search terms used. So, in essence, detail the piloting of the search strategy.

*We have now detailed the piloting of our search strategy. Please see search strategy, lines 123 to 127.*

 Comment 14: Not sure if this will be possible in all cases "For those systematic reviews that combine both studies conducted in intensive care units and hospital ward settings". Please rethink.

*We thank the reviewer for this comment and have now modified this statement to state: we would do this if feasible.*

 Comment 15: You write, "We will exclude systematic reviews which investigated the impact of other staffing metrics, for example, nursing skill mix and nursing work schedules on patient care outcomes". Please explain why.

*We thank the reviewer for this comment. These are other nurse metrics rather than staffing metrics; we have corrected this and stated the focus of our review is on nurse staffing. Please see exposure, lines 94 to 96.*

 Comment 16: This is not clear, "...due to limitations in translation". Do you mean no services available? Or no one on the team can translate? Please think about this a bit more. Are there actually papers that you will really be missing?

*We thank the reviewer for this comment. We have clarified this as a limitation in the translation capacity of the research team.*

 Comment 17: This sentence is consuming "We also conducted initial searches in Prospero to identify ongoing or planned reviews that might relate to our proposed research before undertaking the review.". Was this done to find out if another team is doing an umbrella review? Or if there are ongoing reviews of primary studies? If former, then this should be moved upwards. Either way, this needs to be rephrased for clarity.

*We thank the reviewer for this comment. This was done to identify ongoing or planned reviews and we have now moved this earlier on in our protocol. Please see search strategy, lines 119 to 121.*

 Comment 17: You write, "Pre-review, we will develop a standardised Microsoft Excel form to extract data on each of the identified systematic reviews for our umbrella review". Should this not be available already

*We have now corrected this statement.*

 Comment 18: Should quality assessment come before data extraction?

*We thank the reviewer for this comment and have now corrected this.*

 Comment 19: Please review the content being extracted from the reviews. You write "first author’s name". Is it the name or surname or full name? It is not clear why the metrics such as nurse-to-patient ratios, nurse-to-bed ratios, or nursing hours per patient days need to be extracted from the primary studies. Do you want to maybe review this and fix it from the point of inclusion? Clustered bar charts are also Figures. Is there a reason why this is being specified?

*We thank the reviewer for this comment and have now corrected this to surname. We also plan to extract the staffing metrics from review summary tables of primary articles and if these are not presented, extract them from the cited primary studies. Please see data items section.*

 Comment 20: Clustered bar charts are also Figures. Is there a reason why this is being specified?

*This has been corrected to tables and figures.*

 Comment 21: Please rewrite the discussion to reflect the global nature of this review of reviews. In addition, make a case for why it is important to conduct this review of reviews. What will it show differently compared to what the actual reviews will not show? You can also bring in your dissemination plan (which I suggested should be deleted from ethics above) here.

*This has now been revised and the importance of conducting a review of reviews has been emphasized. Please see discussion lines 175 to 190.*

# Peer review: Reviewer response for version 1

**DOI:** 10.21956/wellcomeopenres.19273.r47865

**Published:** 2022-01-10

**Authors:** Denny John

**Affiliations:** 1 Ramaiah University of Applied Sciences, Bengaluru, Karnataka, India

The authors have used standard guidelines for umbrella reviews, registered with PROSPERO, and also mentioned use of the AMSTAR-2 checklist for assessing quality of systematic reviews. However, for patient reported outcomes some reviews might come from cost-effectiveness studies where the CiCERO might be useful (Mandrik *et al.*, 2021 ^1^).

 The authors are also suggested to use the PRISMA-S checklist during the final review process to report search strategy details.

Is the study design appropriate for the research question?

Yes

Is the rationale for, and objectives of, the study clearly described?

Yes

Are sufficient details of the methods provided to allow replication by others?

Yes

Are the datasets clearly presented in a useable and accessible format?

Yes

Reviewer Expertise:

Evidence synthesis, health technology assessment, public health in developing countries.

I confirm that I have read this submission and believe that I have an appropriate level of expertise to confirm that it is of an acceptable scientific standard.

## References

[1] : Critical Appraisal of Systematic Reviews With Costs and Cost-Effectiveness Outcomes: An ISPOR Good Practices Task Force Report. *Value Health* .24(4) : 10.1016/j.jval.2021.01.002 463-472 10.1016/j.jval.2021.01.002 33840423
